# National Artificial Limb Factory

**Published:** 1919-01

**Authors:** 


**A National Artificial Limb Factory at Gorla Primo near Milan (Italy).** By MM. R. Galleazi and A. Lollini. *Revue Interalliée pour les Mutilés de la Guerre,* April, 1918.

Among the qualities necessary in a working man's substitute limbs the following are noted :

1) The appliance must be easily and solidly fixed to the stump.

2) It must be as simple as possible.

3) It must afford the stump liberty of movement.

4) It must be strong.

5) It must grasp the tools firmly and change them rapidly.

6) The tool must not be fixed rigidly, but must be free to move in all directions necessary during the work.

7) The possibility of using ordinary tools is a great advantage.

An apparatus presenting those qualities is manufactured in the Italian National Prothesis Factory. It is fixed to the stump by means of an artificial fore-arm made up of metallic rings. These rings are not complete circles; their extremities are fixed to an elastic band running through them all. By shortening this band more or less, the rings can be made to embrace the stump tightly. This arm-piece does not slide off, even if heavy weights are carried by means of it. It is easily tolerated by the mutilés, who keep it on for 24 hours without inconvenience.

At its lower extremity is fixed a strong, small, spherical sort of vice for holding the handles of the tools. In fact this vice grasps tightly a metal sphere which is either hooked or screwed to the ordinary work-tools. The sphere can either be fixed rigidly or allowed to move freely into the vice, according to the needs of the work. The opening and closing of the vice is simple and can be easily done by the mutilé himself without the help of his sound hand.

